# No evidence for association between rs10191329 severity locus and longitudinal disease severity in 1813 relapse-onset multiple sclerosis patients from the MSBase registry

**DOI:** 10.1177/13524585241240406

**Published:** 2024-03-21

**Authors:** Maria Pia Campagna, Eva Kubala Havrdova, Dana Horakova, Guillermo Izquierdo, Fuencisla Matesanz, Sara Eichau, Jeannette Lechner-Scott, Bruce V Taylor, Maria-Isabel García-Sanchéz, Antonio Alcina, Anneke van der Walt, Helmut Butzkueven, Vilija G Jokubaitis

**Affiliations:** Department of Neuroscience, School of Translational Medicine, Monash University, Melbourne, VIC, Australia; Department of Neurology and Centre of Clinical Neuroscience, First Faculty of Medicine, Charles University and General University Hospital, Prague, Czech Republic; Department of Neurology and Centre of Clinical Neuroscience, First Faculty of Medicine, Charles University and General University Hospital, Prague, Czech Republic; Hospital Universitario Virgen Macarena, Sevilla, Spain; Instituto de Parasitología y Biomedicina López Neyra, CSIC, Granada, Spain; Hospital Universitario Virgen Macarena, Sevilla, Spain; Department of Neurology, John Hunter Hospital, Newcastle, NSW, Australia; School of Medicine and Public Health, The University of Newcastle, Newcastle, NSW, Australia; Menzies Institute for Medical Research, University of Tasmania, Hobart, TAS, Australia; UGC Neurología, Hospital Universitario Virgen Macarena, Nodo Biobanco del Sistema Sanitario Público de Andalucía, Sevilla, Spain; Instituto de Parasitología y Biomedicina López Neyra, CSIC, Granada, Spain; Department of Neuroscience, School of Translational Medicine, Monash University, Melbourne, VIC, Australia; Department of Neurology, Alfred Health, Melbourne, VIC, Australia; Department of Neuroscience, School of Translational Medicine, Monash University, Melbourne, VIC, Australia; Department of Neurology, Alfred Health, Melbourne, VIC, Australia; Department of Neuroscience, School of Translational Medicine, Monash University, Melbourne, VIC, Australia; Department of Neurology, Alfred Health, Melbourne, VIC, Australia

**Keywords:** Multiple sclerosis, disease severity, genetics

## Abstract

**Background:**

The International Multiple Sclerosis Genetics Consortium and MultipleMS Consortium recently reported a genetic variant associated with multiple sclerosis (MS) severity. However, it remains unclear if these variants remain associated with more robust, longitudinal measures of disease severity.

**Methods:**

We examined the top variant, rs10191329, from Harroud et al.’s study in 1813 relapse-onset MS patients from the MSBase Registry to assess association with longitudinal disease severity.

**Results:**

Our analysis revealed no significant association between rs10191329 genotype and longitudinal binary disease severity (*p* > 0.05).

**Conclusion:**

These findings highlight the complexity of genetic factors mediating long-term MS outcomes and the need for further research.

The International Multiple Sclerosis Genetics Consortium and MultipleMS Consortium (Harroud et al.) recently reported the association of a genetic variant with multiple sclerosis (MS) severity.^1^ This variant, rs10191329 (*DYSF-ZNF638*), was identified in a group of 12,584 European patients and replicated in an additional 9805 with either relapse-onset or primary progressive MS. The study found a small but statistically significant association between rs10191329 and cross-sectional age-related MS severity (ARMSS) scores. Specifically, individuals carrying the A allele at this locus exhibited a 0.089-point higher ARMSS score, a mean of 18.2 years after MS diagnosis (*p* = 3.6 × 10^−9^). Furthermore, homozygous minor allele carriers experienced a faster progression to requirement for a walking aid (an Expanded Disability Status Scale (EDSS) score of 6), with a hazard ratio for progression of 1.22 (95% confidence interval (CI) = 1.09–1.38). In addition to this one replicated variant, the authors identified 11 additional variants that showed suggestive associations (*p* < 10^−5^) with cross-sectional ARMSS scores in the discovery cohort: rs149097173, rs2876767, rs181310516, rs147933117, rs194722, rs12494504, rs112663015, rs9397000, rs61215450, rs4251626 and rs115687581.

It remains uncertain whether these variants maintain their associations with disease severity when using more robust, longitudinal measures of disease severity. We recently published the first genome-wide association study of longitudinal disease severity in relapse-onset MS.^2^ Longitudinally and prospectively collected clinical outcomes data from the MSBase Registry^3^ enabled us to calculate the median ARMSS score of 1813 patients of European ancestry from Australia, Spain and the Czech Republic over a median follow-up of 11.7 years (interquartile range (IQR) = 9.7–15.2) and 21 EDSS scores (IQR = 14–34). This frequency of assessment equates to an EDSS score being documented approximately every 6.7 months for each participant. As previously described,^2^ we tested the association between genotype at >5.9 million single-nucleotide variants (SNVs) and four severity phenotypes: binary longitudinal multiple sclerosis severity score (l-MSSS, that is, mild (*n* = 585) or severe (*n* = 466)), continuous l-MSSS, binary longitudinal ARMSS score (l-ARMSS, that is, mild (*n* = 447) or severe (*n* = 464)), and continuous l-ARMSS. In Jokubaitis et al., we applied linear regression for the continuous variables, adjusted for various factors like the first five principal components, weighted MS genetic risk score and disease-modifying therapy (DMT) use. We conducted a second analysis excluding DMT use as a covariate based on Harroud et al.’s reporting of potential collider bias. We analysed binary outcomes using logistic regression, adjusted for the first five principal components. All analyses were additionally adjusted for demographic variables with a standardised difference greater than 15% between severity extremes. Using these data, we sought to validate the top, replicated variant (rs10191329) from Harroud et al.^1^ using longitudinally measured binary severity outcomes. We further conducted exploratory analyses of the 11 suggestively associated variants (*p* < 10^−5^) from Harroud et al., as well as continuous severity outcomes. Two of these 11 SNVs, rs181310516 and rs12494504, were not present in our datasets. Furthermore, rs149097173, rs112663015 and rs115687581 passed quality control filtering in the binary analysis datasets only. We used *plink v1.9* to identify SNVs that exhibited linkage disequilibrium with the missing SNVs within a 200-kb window. Among these missing SNVs, we could only identify a tagging SNV for rs55787257 (rs112663015, *r*^2^ = 0.56). Our analysis of rs10191329 had 83.8% and 84.8% power to detect an association between genotype (additive) and binary l-ARMSS and l-MSSS at the 0.05 alpha level. We were underpowered to test the association between rs10191329 genotype (additive) and continuous l-ARMSS and l-MSSS outcomes at the 0.05 alpha level (41.8% and 61.4% power, respectively).

Our study did not find an association between rs10191329 genotype and longitudinally defined binary severity outcomes (OR_l-ARMSSbinary_ = 1.04, P_l-ARMSSbinary_ = 0.80; OR_l-MSSSbinary_ = 1.04, P_l-MSSSbinary_ = 0.81) despite having sufficient power to detect such an association. Omitting DMT use from our analyses did not significantly alter our results (OR_l-ARMSSbinary_ = 1.03, P_l-ARMSSbinary_ = 0.82; OR_l-MSSSbinary_ = 1.03, P_l-MSSSbinary_ = 0.80), demonstrating that collider bias was not a major factor in our study. Our exploratory analysis of continuous severity outcomes, and the 11 suggestive SNVs from Harroud et al., also showed no association with longitudinally defined severity outcomes (Tables 1 and 2). However, these results could be attributed to a lack of statistical power. Overall, our findings align with a recent study by Kreft et al.,^4^ which also failed to validate the association between rs10191329 and various disease severity measures, including time to EDSS milestones, ARMSS score, annualised relapse rate and anatomical localisation of relapses. However, Kreft et al. were able to validate the association and direction of effect between longitudinal median ARMSS and MSSS score and rs7289446 and rs868824, respectively, in their prospective and longitudinal cohort of 1455 patients from the South Wales MS Registry. These two variants were the most suggestive SNVs from our work in the MSBase Registry cohort.^2^

**Table 1.** Test statistics for the top 11 suggestively associated variants (*p* < 1e–5) from Harroud et al. (2023) in 1813 longitudinally phenotyped patients from Jokubaitis et al. (2023) – binary outcome measure.

Variant (tagging variant)	Chr	Position (bp)	Gene	Minor allele	MAF	Harroud (2023)^a^		Jokubaitis (2023)											
								Binary outcomes											
								l-ARMSS			l-MSSS			l-ARMSS (collider bias tested)			l-MSSS (collider bias tested)		
						β	*p*	OR	*p* ^b^	Power^c^	OR	*p* ^d^	Power	OR	*p* ^e^	Power	OR	*p* ^f^	Power
rs10191329	2	71676999	*DYSF-ZNF638*	A	0.167	0.09	9.69x10^-9^	1.04	0.80	0.84	1.04	0.81	0.85	1.03	0.82	0.76	1.03	0.80	0.76
rs149097173	1	172370873	*DNM3-PIGC*	T	0.001	0.26	4.07x10^-6^	0.46	0.15	0.01	0.46	0.14	0.01	0.44	0.18	0.01	0.53	0.13	0.01
rs2876767	13	82044107	*-*	C	0.05	-0.14	8.76x10^-8^	0.98	0.94	0.01	1.04	0.83	0.03	0.95	0.82	0.01	1.04	0.87	0.03
rs181310516	1	200217158	*-*	T	NA	-0.35	1.55x10^-7^	NA	NA	NA	NA	NA	NA	NA	NA	NA	NA	NA	NA
rs147933117	3	24926166	*RARB*	A	0.01	0.23	3.40x10^-6^	0.62	0.34	0.04	0.62	0.41	0.04	0.58	0.27	0.05	0.68	0.39	0.03
rs194722	14	69320947	*-*	T	0.20	-0.07	1.39x10^-6^	0.84	0.18	0.14	0.71	0.42	0.80	0.85	0.19	0.12	0.91	0.45	0.04
rs12494504	3	2831218	*CNTN4*	A	0.01	0.21	1.37x10^-6^	NA	NA	NA	NA	NA	NA	NA	NA	NA	NA	NA	NA
rs112663015 (rs55787257^g^)	7	37640233 (37818628)	*-*	A (C)	NA (0.01)	-0.22	4.24x10^-6^	NA (1.29)	NA (0.65)	NA (0.02)	NA (0.72)	NA (0.51)	NA (0.02)	NA (1.15)	NA (0.80)	NA (0.01)	NA (0.69)	NA (0.45)	NA (0.03)
rs9397000	6	170164053	*ERMARD*	T	0.05	-0.13	2.10x10^-6^	1.85	0.01	0.95	1.85	0.17	0.98	1.83	0.01	0.94	1.32	0.18	0.28
rs61215450	6	148550897	*-*	A	0.09	0.09	2.45x10^-6^	1.34	0.09	0.60	1.34	0.54	0.72	1.33	0.09	0.58	1.10	0.55	0.20
rs4251626	14	54875607	*CDKN3*	G	0.22	-0.07	3.52x10^-6^	0.95	0.71	0.01	1.02	0.89	0.86	0.95	0.70	0.01	1.02	0.86	0.86
rs115687581	5	169785565	*KCNIP1*	A	0.03	-0.16	2.94x10^-6^	1.42	0.25	0.17	1.42	0.52	0.23	1.38	0.22	0.14	1.19	0.51	0.18

ARMSS: age-related MS severity score; bp: base pair; b: beta; Chr: chromosome; IMSGC: International MS Genetics Consortium; MAF: minor allele frequency; MSSS: MS severity score; NA: not applicable; OR: odds ratio.

a Discovery cohort statistics.

b Adjusted for: principal components 1–5, % time on therapy since disease onset, weighted genetic risk score, number of DRB1*1501 alleles and imbalanced variables: follow-up time in MSBase (years), symptom duration (years), annualised relapse rate.

c rs10191329 power tested using alpha of 0.05. For suggestive variants, power tested using alpha 0.05/9 (0.006) for binary outcomes.

d Adjusted for: principal components 1–5, % time on therapy since disease onset, weighted genetic risk score, number of DRB1*1501 alleles and imbalanced variables: symptom duration (years), age at most recent visit, number of EDSS scores assessed.

e Adjusted for: principal components 1–5, weighted genetic risk score, number of DRB1*1501 alleles and imbalanced variables: follow-up time in MSBase (years), symptom duration (years), annualised relapse rate.

f Adjusted for: principal components 1–5, weighted genetic risk score, number of DRB1*1501 alleles and imbalanced variables: symptom duration (years), age at most recent visit, number of EDSS scores assessed.

g *r*^2^ = 0.557.

**Table 2.** Test statistics for the top 11 suggestively associated variants (*p* <1e–5) from Harroud et al. (2023) in 1813 longitudinally phenotyped patients from Jokubaitis et al. (2023) – continuous outcome measure.

Variant (tagging variant)	Chr	Position (bp)	Gene	Minor allele	MAF	Harroud (2023)^a^		Jokubaitis (2023)											
								Continuous outcomes											
								l-ARMSS			l-MSSS			l-ARMSS (collider bias tested)			l-MSSS (collider bias tested)		
						β	*p*	β	*p* ^b^	Power^c^	β	*p* ^d^	Power	β	*p* ^e^	Power	β	*p* ^f^	Power
rs10191329	2	71676999	*DYSF-ZNF638*	A	0.167	0.09	9.69x10^-9^	0.08	0.49	0.42	0.42	0.10	0.61	0.08	0.51	0.39	0.11	0.33	0.68
rs149097173	1	172370873	*DNM3-PIGC*	T	0.001	0.26	4.07x10^-6^	NA	NA	NA	NA	NA	NA	NA	NA	NA	NA	NA	NA
rs2876767	13	82044107	*-*	C	0.05	-0.14	8.76x10^-8^	0.07	0.72	0.04	0.04	0.07	0.04	0.08	0.69	0.05	0.09	0.62	0.07
rs181310516	1	200217158	*-*	T	NA	-0.35	1.55x10^-7^	NA	NA	NA	NA	NA	NA	NA	NA	NA	NA	NA	NA
rs147933117	3	24926166	*RARB*	A	0.01	0.23	3.40x10^-6^	0.06	0.52	0.01	0.01	0.10	0.01	-0.64	0.07	0.88	-0.36	0.30	0.31
rs194722	14	69320947	*-*	T	0.20	-0.07	1.39x10^-6^	-0.14	0.18	0.77	0.77	-0.12	0.60	-0.12	0.24	0.60	-0.12	0.25	0.60
rs12494504	3	2831218	*CNTN4*	A	0.01	0.21	1.37x10^-6^	NA	NA	NA	NA	NA	NA	NA	NA	NA	NA	NA	NA
rs112663015 (rs55787257^g^)	7	37640233 (37818628)	*-*	A (C)	NA (0.01)	-0.22	4.24x10^-6^	NA	NA	NA	NA	NA	NA	NA	NA	NA	NA	NA	NA
rs9397000	6	170164053	*ERMARD*	T	0.05	-0.13	2.10x10^-6^	0.31	0.12	0.92	0.92	0.24	0.69	0.32	0.11	0.94	0.22	0.26	0.60
rs61215450	6	148550897	*-*	A	0.09	0.09	2.45x10^-6^	0.11	0.44	0.23	0.23	0.10	0.18	0.13	0.35	0.34	0.10	0.48	0.18
rs4251626	14	54875607	*CDKN3*	G	0.22	-0.07	3.52x10^-6^	-0.05	0.64	0.08	0.08	-0.01	0.01	-0.04	0.72	0.05	0.01	0.95	0.01
rs115687581	5	169785565	*KCNIP1*	A	0.03	-0.16	2.94x10^-6^	NA	NA	NA	NA	NA	NA	NA	NA	NA	NA	NA	NA

ARMSS: age-related MS severity score; bp: base pair; b: beta; Chr: chromosome; IMSGC: International MS Genetics Consortium; MAF: minor allele frequency; MSSS: MS severity score; NA: not applicable; OR: odds ratio.

a Discovery cohort statistics.

b Adjusted for: principal components 1–5, % time on therapy since disease onset, weighted genetic risk score, number of DRB1*1501 alleles and imbalanced variables: follow-up time in MSBase (years), symptom duration (years), annualised relapse rate.

c rs10191329 power tested using alpha of 0.05. For suggestive variants, power tested using alpha of 0.05/6 (0.008) for continuous outcomes.

d Adjusted for: principal components 1–5, % time on therapy since disease onset, weighted genetic risk score, number of DRB1*1501 alleles and imbalanced variables: symptom duration (years), age at most recent visit, number of EDSS scores assessed.

e Adjusted for: principal components 1–5, weighted genetic risk score, number of DRB1*1501 alleles and imbalanced variables: follow-up time in MSBase (years), symptom duration (years), annualised relapse rate.

f Adjusted for: principal components 1–5, weighted genetic risk score, number of DRB1*1501 alleles and imbalanced variables: symptom duration (years), age at most recent visit, number of EDSS scores assessed.

g *r*^2^ = 0.557.

This implies that our inability, along with Kreft et al.’s, to validate rs10191329 may be attributed to differences in cohort and phenotype definition compared to Harroud et al. The former two studies included prospectively and longitudinally ascertained clinical outcomes data, allowing for the calculation of disease severity scores over the entire follow-up period. In contrast, Harroud et al. optimised for sample size, resulting in a much larger cohort with less robust cross-sectionally measured ARMSS scores. It is important to note that predictors of disease progression based on EDSS score measurements, including MSSS and ARMSS scores, are limited by subjective assessment of functional systems. Therefore, until there is an objective biomarker of disability progression, differences in phenotype and cohort characteristics may contribute to the challenges in validating associations across different studies.

Ultimately, Harroud et al.’s study shed light on the genetic factors and tissues that influence MS severity. Their findings complement our own work,^2^ which also identified signals implicating central nervous system function in mediating MS severity outcomes. However, our results in the present replication study caution that genetic factors mediating long-term MS outcomes are complex, require further study and are not yet ready for clinical translation.
